# Estimated Medicaid Costs Associated with Hepatitis A During an Outbreak — West Virginia, 2018–2019

**DOI:** 10.15585/mmwr.mm7008a2

**Published:** 2021-02-26

**Authors:** Samantha J. Batdorf, Megan G. Hofmeister, Tamara C. Surtees, Erica D. Thomasson, Shannon M. McBee, Nathan J. Pauly

**Affiliations:** ^1^Bureau for Public Health, West Virginia Department of Health and Human Resources; ^2^Division of Viral Hepatitis, National Center for HIV/AIDS, Viral Hepatitis, STD, and TB Prevention, CDC; ^3^Immunization Services Division, National Center for Immunization and Respiratory Diseases, CDC; ^4^Division of State and Local Readiness, Center for Preparedness and Response, CDC; ^5^Medicaid Research and Evaluation, West Virginia University, Morgantown, West Virginia.

Hepatitis A is a vaccine-preventable disease caused by the hepatitis A virus (HAV). Transmission of the virus most commonly occurs through the fecal-oral route after close contact with an infected person. Widespread outbreaks of hepatitis A among persons who use illicit drugs (injection and noninjection drugs) have increased in recent years (*1*). The Advisory Committee on Immunization Practices (ACIP) recommends routine hepatitis A vaccination for children and persons at increased risk for infection or severe disease, and, since 1996, has recommended hepatitis A vaccination for persons who use illicit drugs (*2*). Vaccinating persons who are at-risk for HAV infection is a mainstay of the public health response for stopping ongoing person-to-person transmission and preventing future outbreaks (*1*). In response to a large hepatitis A outbreak in West Virginia, an analysis was conducted to assess total hepatitis A–related medical costs during January 1, 2018–July 31, 2019, among West Virginia Medicaid beneficiaries with a confirmed diagnosis of HAV infection. Among the analysis population, direct clinical costs ranged from an estimated $1.4 million to $5.6 million. Direct clinical costs among a subset of the Medicaid population with a diagnosis of a comorbid substance use disorder ranged from an estimated $1.0 million to $4.4 million during the study period. In addition to insight on preventing illness, hospitalization, and death, the results from this study highlight the potential financial cost jurisdictions might incur when ACIP recommendations for hepatitis A vaccination, especially among persons who use illicit drugs, are not followed (*2*).

Historically, hepatitis A infections have been rare in West Virginia, with an average of eight cases reported annually to the state Bureau for Public Health during 2007–2013 (*3*). Since March 2018, West Virginia has experienced a series of hepatitis A outbreaks, primarily among persons who use illicit drugs (*4*). As of February 2020, a total of 2,702 outbreak-related cases had been reported; approximately two thirds of patients reported illicit drug use, approximately one half of the outbreak-related patients were hospitalized, and 23 deaths were reported (*4*). The cost of West Virginia’s hepatitis A outbreak has not been previously quantified.

Paid claims for West Virginia Medicaid beneficiaries with a diagnosis of hepatitis A* during January 1, 2018–July 31, 2019 were examined. These data were extracted from the West Virginia Bureau for Medical Services’ Data Warehouse on request by the West Virginia Bureau for Public Health. A total of 64 patients who had a claim with a procedure code for hepatitis A vaccination^†^ during the study period were excluded (*5*) (Figure). Pharmacy claims were also excluded because no specific pharmacologic treatment exists for hepatitis A (*6*). Total hepatitis A–related medical costs were assessed in three of the following ways: 1) scenario 1, in which costs associated with claims that had any diagnosis (i.e., primary or secondary diagnosis) of hepatitis A were summed to obtain the least conservative estimate of hepatitis A–related costs, 2) scenario 2, in which costs associated with claims that had a primary diagnosis of hepatitis A were summed to obtain a more conservative cost estimate, and 3) scenario 3, in which costs associated with inpatient hospital claims that had both a primary diagnosis of hepatitis A and a diagnosis-related group (DRG) code indicating disorders of the liver^§^ were summed to obtain the most conservative cost estimate. Hepatitis A–related costs were also measured for the subgroup of patients in each scenario with comorbid substance use disorder. Persons who had at least one claim with a primary or secondary diagnosis related to substance use disorder (excluding nicotine- or alcohol-related substance use disorders^¶^) during the study period were classified as having comorbid substance use disorder. Analyses were conducted using SAS (version 9.4; SAS Institute). This study was deemed not to be human subjects research by CDC and was exempt from Institutional Review Board review; the study was reviewed by CDC and conducted consistent with applicable federal law and CDC policy.**

**FIGURE:**
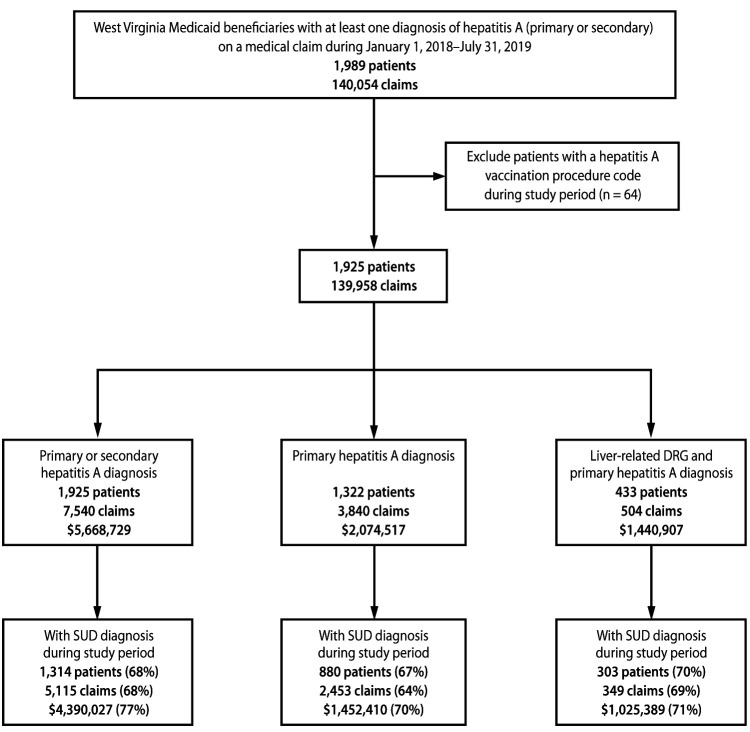
Inclusion criteria for analysis of Medicaid beneficiaries with at least one hepatitis A diagnosis* on a medical claim — West Virginia, January 1, 2018–July 31, 2019^†^ **Abbreviations**: DRG = diagnosis-related group; SUD = substance use disorder. * Direct clinical costs are shown for each hepatitis A diagnosis/SUD group. ^†^ SUD diagnoses exclude those related to alcohol or nicotine.

A total of 1,989 Medicaid beneficiaries with a diagnosis of hepatitis A (primary or secondary) were identified; 1,925 patients met study inclusion criteria for scenario 1, 1,322 patients met the criteria for scenario 2, and 433 patients met the criteria for scenario 3 (Figure). The median age of the 1,925 patients in scenario 1 was 37 years (range = 3–83 years) and the majority were male (54%) (Table 1). Approximately two thirds of study patients had a comorbid substance use disorder diagnosis on a claim at some point during the study period.

**TABLE 1:** Demographic and risk factor characteristics of Medicaid beneficiaries with a hepatitis A diagnosis* — West Virginia, January 1, 2018–July 31, 2019

Characteristic	No. (%)		
	Scenario 1^†^: primary or secondary hepatitis A diagnosis	Scenario 2^§^: primary hepatitis A diagnosis	Scenario 3^¶^: liver-related DRG and primary hepatitis A diagnosis
**Overall sample**			
**Unique patients**	**1,925**	**1,322**	**433**
Median age, yrs (range)	37 (3–83)	37 (3–83)	38 (18–68)
Male	1,036 (54)	738 (56)	259 (60)
Female	889 (46)	584 (44)	174 (40)
Nonalcohol or nicotine SUD patients during the study period	1,314 (68)	880 (67)	303 (70)
**Subgroup with nonalcohol or nicotine SUD during the study period**			
**Unique patients**	**1,314**	**880**	**303**
Median age, yrs (range)	35 (13–71)	35 (13–71)	35 (18–66)
Male	735 (56)	512 (58)	189 (62)
Female	579 (44)	368 (42)	114 (38)

**Abbreviations**: DRG = diagnosis-related group; SUD = substance use disorder.

* Hepatitis A–related clinical costs were assessed in three ways.

^†^ Scenario 1: costs associated with medical claims that had a primary or secondary diagnosis of hepatitis A were summed to obtain the least conservative estimate of hepatitis A–related costs.

^§^ Scenario 2: costs associated with medical claims that had a primary diagnosis of hepatitis A were summed to obtain a more conservative cost estimate.

^¶^ Scenario 3: costs associated with inpatient hospital claims that had both a primary diagnosis of hepatitis A and a diagnosis-related group code indicating disorders of the liver were summed to obtain the most conservative cost estimate.

Total hepatitis A–related clinical costs among all Medicaid beneficiaries with a diagnosis of hepatitis A ranged from $1,440,907 (scenario 3) to $5,668,729 (scenario 1) (Table 2). Among the 1,314 patients with a comorbid substance use disorder diagnosis, the total hepatitis A–related clinical costs ranged from $1,025,389 (scenario 3) to $4,390,027 (scenario 1) (Table 2).

**TABLE 2:** Hepatitis A–related Medicaid direct clinical costs* — West Virginia, January 1, 2018–July 31, 2019

Characteristic	Scenario 1^†^: primary or secondary hepatitis A diagnosis	Scenario 2^§^: primary hepatitis A diagnosis	Scenario 3^¶^: liver-related DRG and primary hepatitis A diagnosis
**Overall sample**			
No. of unique patients	1,925	1,322	433
Total hepatitis A–related direct clinical costs, $	5,668,729	2,074,517	1,440,907
**Subgroup with nonalcohol or nicotine SUD during study period**			
No. of unique patients	1,314	880	303
Total hepatitis A–related direct clinical costs, $	4,390,027	1,452,410	1,025,389

**Abbreviations**: DRG = diagnosis-related group; SUD = substance use disorder.

* Hepatitis A–related clinical costs were assessed in three ways.

^†^ Scenario 1: costs associated with medical claims that had a primary or secondary diagnosis of hepatitis A were summed to obtain the least conservative estimate of hepatitis A–related costs.

^§^ Scenario 2: costs associated with medical claims that had a primary diagnosis of hepatitis A were summed to obtain a more conservative cost estimate.

^¶^ Scenario 3: costs associated with inpatient hospital claims that had both a primary diagnosis of hepatitis A and a diagnosis-related group code indicating disorders of the liver were summed to obtain the most conservative cost estimate.

## Discussion

This analysis identified 1,925 West Virginia Medicaid beneficiaries whose medical claims included a hepatitis A diagnosis during January 1, 2018–July 31, 2019, and met the study inclusion criteria. During the study period, the total expenditure for medical claims with a hepatitis A diagnosis exceeded $5.6 million, including approximately $1.4 million spent on hepatitis A–related inpatient hospital admissions alone. Illicit drug use is a known risk factor for HAV infection (*1*); claims for 68% of persons in this study included a substance use disorder diagnosis. The total hepatitis A–related costs for this group was approximately $4.4 million during the study period.

The findings in this report are subject to at least four limitations. First, administrative claims data used for this analysis were generated for reimbursement purposes, not research. One previous study assessing the usefulness of claims data for hepatitis surveillance reported higher rates of false positive diagnoses in claims data relative to other data sources (*7*). Second, costs were assumed to be directly attributable to the hepatitis A diagnosis on the claims. Presumably, most services were directly related to the primary diagnosis recorded on the claim; however, this might not always have been the case. The three scenarios described previously were used to mitigate this limitation. Third, the hepatitis A–related costs assessed in this analysis incorporated only direct clinical costs to the West Virginia Medicaid agency for persons with a diagnosis of hepatitis A in the context of an outbreak. These are conservative cost estimates that do not include expenses associated with the public health outbreak response, productivity loss, other indirect costs, or direct costs from pharmacy claims. In addition, by focusing on direct clinical costs to the West Virginia Medicaid agency, this analysis did not consider hepatitis A–related clinical costs borne by private insurers, Medicare, and other payers, or account for costs borne from treatment of the uninsured. Thus, the hepatitis A–related clinical costs presented in this report likely underestimate the total clinical costs of West Virginia’s outbreak. Finally, this analysis was limited to the West Virginia Medicaid population; therefore, the results might not be directly generalizable to other states or demographic groups.

The large hepatitis A outbreak in West Virginia has acutely affected the state’s Medicaid program. The costs associated with hepatitis A clinical care alone during a person-to-person outbreak are substantial. The results presented in this report suggest that the West Virginia Medicaid agency incurred a minimum of $1.4 million in costs directly associated with the first 19 months of this outbreak. Although improving, this outbreak is ongoing as of February 2021 and has resulted in hospitalizations for approximately one half of persons with cases of HAV and 23 reported deaths. In addition to insight on preventing illness, hospitalization, and death, the results from this study highlight the potential financial cost jurisdictions might incur when ACIP recommendations for hepatitis A vaccination, especially among persons who use illicit drugs, are not followed (*2*).

**Summary**

**What is already known about this topic?**

Widespread outbreaks of hepatitis A among persons who use illicit drugs (injection and noninjection) have increased in recent years. Hepatitis A is a vaccine-preventable disease.

**What is added by this report?**

During January 1, 2018–July 31, 2019, hepatitis A–related clinical costs among West Virginia Medicaid beneficiaries ranged from $1.4 million to $5.6 million. Among those with a substance use disorder diagnosis, costs ranged from $1.0 million to $4.4 million.

**What are the implications for public health practice?**

In addition to insight on preventing illness, hospitalization, and death, the results from this study highlight the potential financial cost jurisdictions might incur when Advisory Committee on Immunization Practices recommendations for hepatitis A vaccination, especially among persons who use illicit drugs, are not followed.

## Footnotes

* Hepatitis A patients were identified using the following *International Classification of Diseases, Tenth Revision* (ICD-10), diagnostic codes: B150 and B159.

† American Medical Association, Current Procedural Terminology, vaccine procedures codes 90632, 90633, 90634, 90636, and 90730.

§ *International Classification of Diseases, Tenth Revision, Clinical Modification*, liver-related diagnosis-related groups 441, 442, and 443.

¶ ICD-10*/Diagnostic and Statistical Manual of Mental Disorders, Fifth Edition*, substance use disorder diagnosis codes (excluding nicotine- and alcohol-related codes): F11.1, F11.2, F11.9, F12.1, F12.2, F12.9, F13.1 F13.2, F13.9, F14.1, F14.2. F14.9, F15.1, F15.2, F15.9, F16.1, F16.2, F16.9, F18.1, F18.2, F18.9, F19.1, F19.2, F19.9, F55[.0-.4], F55.8, O35.5, O99.3, P04.4, P96.1, P96.2, T40.0 , T40.1, T40.5, T40[0.7-0.9].

** 45 C.F.R. part 46.102(l)(2), 21 C.F.R. part 56; 42 U.S.C. Sect. 241(d); 5 U.S.C. Sect. 552a; 44 U.S.C. Sect. 3501 et seq.
